# Using NS1 Flavivirus Protein Microarray to Infer Past Infecting Dengue Virus Serotype and Number of Past Dengue Virus Infections in Vietnamese Individuals

**DOI:** 10.1093/infdis/jiaa018

**Published:** 2020-01-22

**Authors:** Tran Thi Nhu Thao, Erwin de Bruin, Huynh Thi Phuong, Nguyen Ha Thao Vy, Henk-Jan van den Ham, Bridget A Wills, Nguyen Thi Hanh Tien, Huynh Thi Le Duyen, Dinh The Trung, Stephen S Whitehead, Maciej F Boni, Marion Koopmans, Hannah E Clapham

**Affiliations:** 1 Viroscience Department, Erasmus University of Rotterdam, Rotterdam, the Netherlands; 2 Oxford University Clinical Research Unit, Wellcome Trust Asia Program, Ho Chi Minh City, Vietnam; 3 Institute for Virology and Immunology, University of Bern, Bern, Switzerland; 4 Centre for Tropical Medicine and Global Health, Nuffield Department of Medicine, University of Oxford, Oxford, United Kingdom; 5 Laboratory of Viral Diseases, National Institutes of Health, Bethesda, Maryland, USA; 6 Center for Infectious Disease Dynamics, Department of Biology, Pennsylvania State University, University Park, Pennsylvania, USA

**Keywords:** dengue, flaviviruses, seroepidemiology, serology, transmission

## Abstract

**Background:**

In recent years, researchers have had an increased focus on multiplex microarray assays, in which antibodies are measured against multiple related antigens, for use in seroepidemiological studies to infer past transmission.

**Methods:**

We assess the performance of a flavivirus microarray assay for determining past dengue virus (DENV) infection history in a dengue-endemic setting, Vietnam. We tested the microarray on samples from 1 and 6 months postinfection from DENV-infected patients (infecting serotype was determined using reverse-transcription polymerase chain reaction during acute, past primary, and secondary infection assessed using plaque reduction neutralization tests 6 months postinfection).

**Results:**

Binomial models developed to discriminate past primary from secondary infection using the protein microarray (PMA) titers had high area under the curve (0.90–0.97) and accuracy (0.84–0.86). Multinomial models developed to identify most recent past infecting serotype using PMA titers performed well in those with past primary infection (average test set: κ = 0.85, accuracy of 0.92) but not those with past secondary infection (κ = 0.24, accuracy of 0.45).

**Conclusions:**

Our results suggest that the microarray will be useful in seroepidemiological studies aimed at classifying the past infection history of individuals (past primary vs secondary and serotype of past primary infections) and thus inferring past transmission intensity of DENV in dengue-endemic settings. Future work to validate these models should be undertaken in different transmission settings and with samples later after infection.

## Teaser

The results of testing serological samples from an endemic setting with flavivirus protein microarray assays can be used to infer whether individuals have had 1 or 2 past dengue infections and what the infecting serotype was after 1 infection.

Flaviviruses such as Japanese encephalitis, yellow fever, Zika virus (ZIKV), and dengue virus (DENV), transmitted by vectors, are responsible for the most common viral human diseases in many parts of the world [1]. Dengue has a major and expanding economic and health burden worldwide, especially in tropical regions. Recent estimates suggest that there are approximately 390 million dengue infections annually, 70% of which are estimated to be in Asia [2]. In Vietnam, dengue is endemic, especially in the southern areas, with 1.50–2.75 million symptomatic infections estimated per year [2]. Seasonal outbreaks are observed mostly in the southern part of the country during the rainy season from June to December [3].

Dengue virus infection can be asymptomatic or elicit a spectrum of clinical symptoms ranging from an uncomplicated febrile illness to dengue with warning signs or severe dengue, including the potentially fatal dengue shock syndrome [4]. There are 4 DENV serotypes (DENV1 to DENV4) that are further classified into genotypes. A first (primary) infection with dengue is unlikely to result in complications and provides lifelong immunity to this serotype. After a period of cross-protection, a second infection with a different serotype (a secondary infection) is more likely to be severe due the phenomenon of antibody-dependent enhancement. In each of these time periods, only a proportion (18%–50%) of infected individuals will develop symptoms [5], and only a small proportion of symptomatic patients will have more severe disease and seek care. This means that using information on cases to understand transmission intensity, and therefore for planning future interventions, leads to underestimation of the population attack rate. Therefore, seroepidemiological studies or serosurveys can provide a useful tool to understand past DENV transmission intensity [6–9]. For this reason, serosurveys for DENV were part of the first World Health Organization (WHO) recommendations for Dengvaxia use, which suggested that serosurveys should be used to decide where vaccination could be used [10, 11]. These guidelines have since been superseded by guidelines to only use the vaccine in dengue-seropositive individuals; however, serological studies may still have a use to determine where and at what age testing for seropositivity should occur [12].

There is immunological cross-reactivity among the flaviviruses [13], which complicates the interpretations of serological data with regard to past infection and current protection for all flaviviruses [14, 15]. For DENV, finding an inexpensive, simple, reliable, and high-throughput serological assay that gives interpretable results, taking into account this cross-reactivity, is vital for seroepidemiological studies. Immunoglobulin (Ig)G indirect enzyme-linked immunosorbent assay (ELISA) (as suggested in WHO serosurveys guidelines [11]) and plaque reduction neutralization tests (PRNTs) have been widely used for detecting past exposure. Currently available IgG indirect ELISA assays are relatively cheap, easy to use, and often used for serosurveys, but they cannot determine previous infecting serotype and (currently) are not used to determine whether an individual has been infected once or more than once in the past. The PRNT provides better discrimination, with more DENV specificity, some serotype specificity, and the ability to discriminate single from multiple exposures. Therefore, PRNT has been widely used as the gold standard in serological surveys and vaccine studies. It is also possible to perform PRNT against multiple flaviviruses in multiple experiments, although the reproducibility is thought to be low, with high demands on time and technical expertise from laboratory staff. In addition, heterogeneity between PRNT can be introduced by many other factors such as virus strain, cell line, viral maturation state, and time and temperature of incubation [16]. By contrast, a novel technique, the flavivirus protein microarray (PMA) initially introduced by Cleton et al [17], uses the NS1 protein antigen to detect IgG antibodies to a range of flaviviruses in a highly standardized, high-throughput manner. In previous work to develop and validate this PMA, the authors tested serum samples collected from recently infected but previously flavivirus-naive travelers where they showed good discrimination between flaviviruses [17].

To determine the viability of using the microarray for seroepidemiological studies in dengue-endemic settings, where many individuals will have multiple infections, we tested the assay on follow-up samples from individuals with known infection histories. Using these results, we built models to predict subjects’ infection histories.

## MATERIALS AND METHODS

### Serum Samples

During the 2009–2010 and 2011–2012 transmission seasons, a subgroup of participants already enrolled in studies of suspected dengue (both hospital-based and community-based) coordinated by the Hospital for Tropical Diseases in Ho Chi Minh City (HCMC), Vietnam, were asked to participate in this study [18]. Individuals in whom DENV infection had been confirmed by reverse-transcription polymerase chain reaction (RT-PCR) as part of the initial study were approached, and those who agreed to participate provided additional blood samples at 2 time points after the acute infection: 2–4 weeks (follow-up 1) and 6 months (follow-up 2) [18]. Plaque reduction neutralization test (PRNT)60 was performed on the 6-month follow-up samples at the US National Institutes of Health as described previously [19, 20]. A PRNT60 titer is the reciprocal of the dilution at which a 60% reduction in the number of plaques caused by viral infection in a cell monolayer is observed. In this study, an infection was categorized as primary if the PRNT60 titer to the infecting DENV serotype was ≥20 and the titers to all other DENV serotypes were <20. An infection was categorized as secondary if the PRNT60 titer to the infecting DENV serotype was ≥20 and the titer to at least 1 of the remaining serotypes was either (1) ≥40 or (2) greater than or equal to the titer against the infecting serotype [18]. Cases that did not meet these criteria were classified as indeterminate. As described in the previous publication [18], samples from at least 3 days during the acute illness episode were also tested using IgG and IgM capture and IgG indirect ELISAs. Using these ELISA titers, algorithms were developed that were able to successfully predict primary or secondary infection on any acute illness day, with different cutoffs depending on the day [18]. We selected samples from 184 individuals from this study to test for the results reported here. To ensure that we had as much data as possible from each group (infecting serotype and immune status), this included all of the individuals infected by DENV3 and all primary infections with DENV2 and DENV4 (due to small numbers in these groups [18]) and then a selection from the remaining groups to ensure all groups were represented. As sensitivity analysis, we also resampled the DENV2–4 infections to have a dataset that had data equally distributed across the different serotypes and immune status classifications. This study, and the initial acute illness studies, was approved by the ethics committee of the Hospital of Tropical Diseases HCMC.

### Flavivirus Protein Microarrays 

Flavivirus PMAs were printed at the Viroscience Department (Erasmus Medical Center) as previously described [21]. Multiplex serology to screen for IgG antibodies was performed on serum samples in biosafety level 2 laboratory conditions, as described previously [17, 21]. Inactivated serum samples were serially diluted into 4-fold dilutions starting from 1:20 to 1:1280 using Blocker BLOTTO Blocking Buffer (Invitrogen) containing 0.1% Surfact-Amp (Thermo Fisher Scientific). Samples from after secondary infections that had saturated DENV titers were further diluted by 4-fold from 1:1280 to 1:81 920. The positive control (inactivated pooled sera from patients infected with DENV serotypes 1 to 4) was diluted into 8 dilutions from 1:160 to 1:81 920 (Supplementary Figure S1). A goat antihuman IgG conjugated with Alexa Fluor 647 was used as secondary antibody. After drying, the slides were scanned using a PowerScanner (Tecan).

For each sample, the fluorescent signals were read and used to calculate a single PMA titer by fitting a 4-parameter log-logistic curve using R [22], with 3000 and 65 535 as the lower limit and upper limit of detection, respectively. Antibody titer against an antigen was defined as the serum concentration that corresponded to the inflection point (50% effective concentration) on the fitted curve, as described in [21]. We corrected for variations based on the positive control included on each microarray slide (Supplementary Figure S2). In this analysis, the results of titers against DENVs 1–4 and ZIKVs, West Nile virus (WNV), and St. Louis encephalitis virus (SLEV) were used, because we were readily able to normalize these titers using the positive control. Log_2_-transformed antibody titers were used for analysis.

### Statistical Analysis

We performed all analyses in R v3.1 and v5.1 [22]. User-defined R code is included in Supplementary Information. Using the Mann-Whitney test, we compared antibody titers to each flavivirus antigen on the array at each time point and changes in these responses between follow-up time points between those who had experienced (1) 1 versus more than 1 past infections (primary vs secondary) and (2) between different serotypes of most recent infecting serotype. We also performed multivariable linear regressions to assess the impact of covariates on antibody titers.

Using the microarray titers of the samples at 2–4 weeks and 6 months after infection as well as age as the input variables, we developed (1) binomial models to predict whether an individual had experienced 1 or more than 1 past infections (defined by immune status as determined by PRNT results at 6 months postinfection as described above) and (2) multinomial models to predict most recent past infecting serotype, as determined by RT-PCR results from the most recent acute infection. We included age because it is a readily available covariate that will be available for such epidemiological data, and often no other covariates are available. For model selection, we used Akaike’s information criterion (AIC). We used *k*-fold cross-validation for out-of-sample validation to avoid overfitting (*k *= 5 for binomial logistic models, and *k *= 3 for multinomial logistic models). We assessed (1) the binomial model performance using the area under the curve (AUC) [23, 24] and accuracy (the proportion of samples with outcome correctly identified) and (2) the multinomial logistic model using Cohen’s kappa-statistic [24] and accuracy. For those individuals classified as having more than 1 past exposure (secondary), we were able to compare the models to predict the most recent infecting serotype at 6 months developed using PMA data to models using PRNT titers. To assess the outcome of the assay and methods on infections classified as indeterminate by PRNT, we applied the immune status-discriminating models and then the serotype-predicting models.

## RESULTS

A total of 368 serum samples from 184 patients were tested using the PMAs. After excluding 16 samples due to a missing control or to no reaction having taken place on the whole slide, 176 individuals were included in this analysis. On the basis of the PRNT60 data, 55 patients were classified as having experienced a primary infection, 86 were classified as having experienced a secondary infection, and in 35 cases immune status was indeterminate (Table 1). Dengue virus 1 dominated in the primary cases (closely followed by DENV2); DENV1, -2, and -4 dominated in the secondary cases; and DENV2 dominated in the indeterminate group (Table 1).

**Table 1.** Descriptions of Population Characteristics in the Two Studies Grouped by Whether Classified by PRNT and Acute IgG and IgM as Having Experience One, Two, or Unknown (Indeterminate) Number of Past Infections

PRNT Classification	One Past Infection (Primary)	Two or More Past Infections (Secondary)	Indeterminate Infections
	(n = 55 Subjects)	(N = 86 Subjects)	(N = 35 Subjects)
Age, median (IQR)	13 (11–17)	14 (11–19)	14 (12–20)
Gender, female (%)	13 (24%)	41 (47%)	12 (17%)
Day of follow-up 1, median (IQR)	19 (16–31)	18 (16–30)	17 (16–31)
Day of follow-up 2, median (IQR)	217 (202–244)	200 (183–219)	215 (201–240)
DENV1 PCR-positive in acute infection	28	27	6
DENV2 PCR-positive in acute infection	17	26	15
DENV3 PCR-positive in acute infection	7	12	5
DENV4 PCR-positive in acute infection	3	21	9

Abbreviations: DENV, dengue virus; Ig, immunoglobulin; IQR, interquartile range; PCR, polymerase chain reaction; PRNT, plaque reduction neutralization test.

### Differences in Antibody Profiles After Primary and Secondary Infections and After Infection With the Different Serotypes

**Figure 1.**
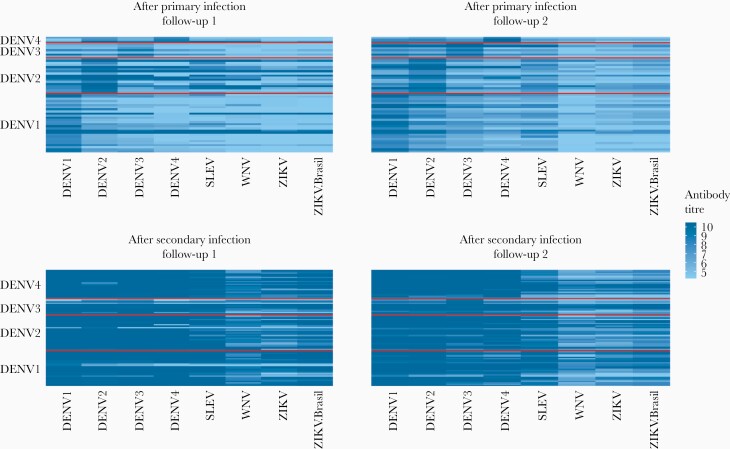
Heatmaps illustrating antibody profiles of individuals (rows) after having experienced a dengue virus (DENV)1–4 infection (row labels). The panels are stratified by immune status (as determined by plaque reduction neutralization test) and follow-up time points. Each row is an individual, and the color indicates the magnitude of the response to each antigen (column labels). SLEV, St. Louis encephalitis virus; WNV, West Nile virus; ZIKV, Zika virus.

**Figure 2.**
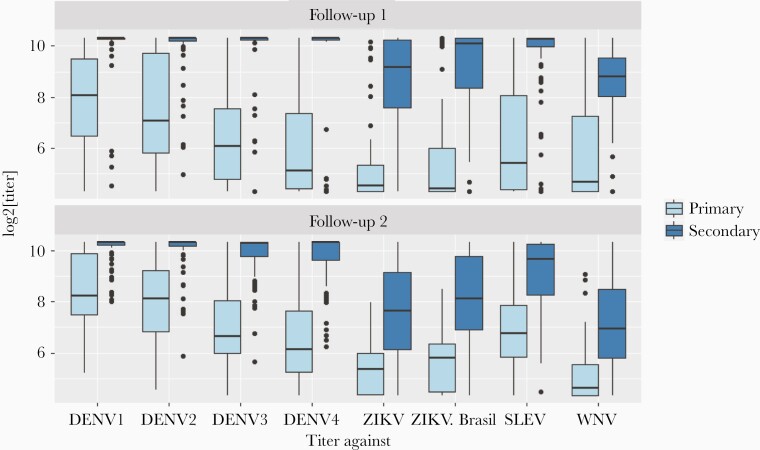
Distributions of microarray antibody titers against all antigens at follow-up time points 1 and 2 after experiencing 1 vs 2 or more infections (as determined by plaque reduction neutralization test and acute immunoglobulin [Ig]G and IgM). Boxplots show medians and interquartile ranges. Highest dilution tested by protein microarray for displayed titers are 10.3 (dilution of 1280). DENV, dengue virus; SLEV, St. Louis encephalitis virus; WNV, West Nile virus; ZIKV, Zika virus.

**Figure 3.**
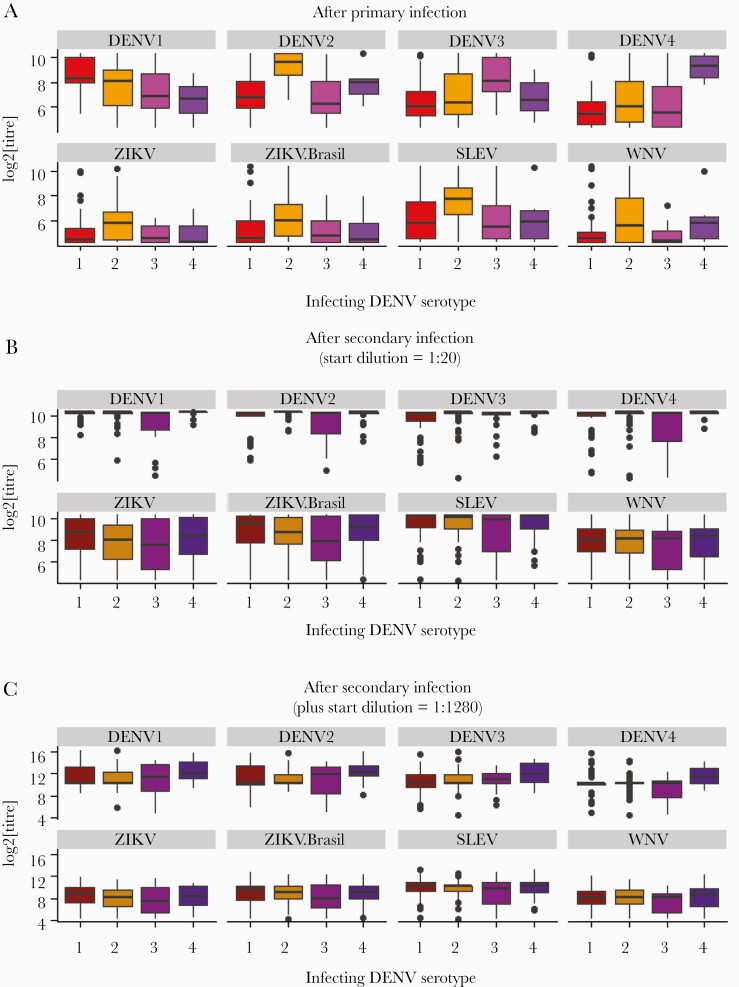
Distributions of microarray antibody titers to each antigen grouped by most recent past infecting serotype after (A) 1 infection, (B) 2 or more infections, and (C) 2 or more infections, but including samples tested again with starting dilution 1:1280. DENV, dengue virus; SLEV, St. Louis encephalitis virus; WNV, West Nile virus; ZIKV, Zika virus.

Antibody titers against DENV antigens were higher after a secondary infection than after a primary infection (Figures 1 and 2 and Supplementary Table S2). This pattern is discernible at both follow-up time points (Mann-Whitney test, *P* < .01 for all serotypes). After primary infection, there is a slight increase in antibody titers from follow-up time point 1 to 2; however, these changes are small and nonsignificant (Mann-Whitney test, *P* > .2 for all serotypes) (Figure 2, Supplementary Figure S3, and Supplementary Table S2). After a primary infection, the antibody titer against the infecting DENV serotype was higher than that against any of the noninfecting serotypes (Figure 3A and Supplementary Table S2). After most secondary infections, there was a slight decrease from follow-up time point 1 to 2: Mann-Whitney test *P* > .1 for DENV1 and DENV2, *P* < .05 for DENV3 and DENV4, with mean log_2_ titers changing from 9.95 to 9.67 for DENV3 and 9.85 to 9.67 for DENV4 (Figure 2, Supplementary Figure S3, and Supplementary Table S2). After a secondary infection, there was no clear pattern of antibody titers according to the most recent infecting serotype, because the antibody titers were uniformly high across all 4 dengue serotypes (Figure 3B and C, and Supplementary Table S2).

Cross-reactive titers to non-DENV antigens were higher after secondary compared with after primary infection. After primary infection, the highest cross-reactivity was observed after DENV2 infections (Figure 3A). Even after a secondary infection, the titers for non-DENV antigens were lower than those for DENV antigens (Figures 1–3).

### Model Selection and Performance

The best models for predicting immune status (past primary vs secondary infections) included DENV3 and DENV4 titers as well as ZIKV, SLEV, and WNV (Table 2 and Supplementary Table S3). The best model for the recent (PCR-confirmed) acute illness serotype included all DENV titers plus SLEV, ZIKV, and age (Table 2 and Supplementary Table S3). These models reliably distinguished primary from secondary infection (Table 3 and Supplementary Tables S6 and S7); models A–C all have high AUC and a high proportion of correctly classified cases (AUC, 0.90–0.97; accuracy, 0.84–0.86) in the 5-fold cross-validation. For determining most recent past infecting serotype, separate models were needed for primary and secondary infections; however, within each immune status group, the same models performed best at each time point and at both time points together (models D and E). The selected models performed well after primary infection (model D [Table 4 and Supplementary Tables S8 and S9], average test set κ = 0.85, accuracy of 0.92), but not after secondary infection (model E), even when including the additional dilutions tested (model F) ([Table 4] models E and F, average test set κ = 0.24 and 0.25, accuracy of 0.45 and 0.43). After primary infection, model performance was the same at both time points (data not shown); however, after secondary infections, model performance improved from time point 1 to 2 (average model performance at time point 1, accuracy of 0.31 and κ = 0.08 vs time point 2, accuracy of 0.47 and κ = 0.27). Age was included in the best predicting model for the infecting serotype model for secondary infections. Because the age of the cohort is limited, we wanted to assess how well this model would do without age. The model performed slightly worse without age: accuracy of 0.39, κ = 0.14.

**Table 2.** AIC-Based Selected Models

Model (Data)	Outcome	Predictors
A (follow-up 1)	1 (primary) vs 2 or more past infections (secondary)	DENV3 + DENV4 + ZIKV + SLEV
B (follow-up 2)	1 (primary) vs 2 or more past infections (secondary)	DENV3 + DENV4 + ZIKV
C (both follow-ups)	1 (primary) vs 2 or more past infections (secondary)	DENV3 + DENV4 + ZIKV + SLEV + WNV
D (primary, both follow-ups)	Infecting DENV serotype	DENV1 + DENV2 + DENV3 + DENV4
E (secondary, both follow-ups)	Infecting DENV serotype	DENV1 + DENV2 + DENV3 + SLEV + ZIKV + Age
F (secondary, both follow-ups, including higher dilutions)	Infecting DENV serotype	DENV1 + DENV2 + DENV3 + DENV4 + SLEV + Age

Abbreviations: AIC, Akaike’s information criterion; DENV, dengue virus; SLEV, St. Louis encephalitis virus; WNV, West Nile virus; ZIKV, Zika virus.

**Table 3.** Average Performance of Immune Status-Discriminating Models in 5-Fold Cross-Validation

Model	AUC (95% CI)	Accuracy
Model A (follow-up point 1) (141 observations: 55 primary cases and 86 secondary cases)	0.90 (0.83–0.96)	0.84
Model B (follow-up point 2) (141 observations: 55 primary cases and 86 secondary cases)	0.95 (0.92–0.98)	0.86
Model C (both follow-up points) (282 observations: 55 primary cases and 86 secondary cases)	0.93 (0.90–0.96)	0.85

Abbreviations: AUC, area under the curve; CI, confidence interval.

**Table 4.** Average Performance of Serotype-Identifying Models in 3-Fold Cross-Validation

Model	Kappa	Accuracy
Model D (primary infections) (110 observations from 28 DENV1, 17 DENV2, 7 DENV3, 3 DENV4 infected individuals)	0.87	0.92
Model E (secondary infections) (172 observations from 27 DENV1, 26 DENV2, 12 DENV3, 21 DENV4 infected individuals)	0.24	0.45
Model F (secondary infections with higher dilution titers for those saturated) (172 observations from27 DENV1, 26 DENV2, 12 DENV3, 21 DENV4 infected individuals)	0.25	0.43

Abbreviations: DENV, dengue virus.

In the resampled dataset, the best models remained similar; however, in some iterations, the model for immune status including DENV1 (the second best fitting model previously) fitted equally well (by AIC and AUC). For predicting the infecting serotypes, the models on the resampled dataset did slightly better after a primary infection (average test set accuracy of 0.98 and κ = 0.96) and slightly worse after a secondary infection (average test set accuracy of 0.39 and κ = 0.19).

### Comparing Protein Microarrays Versus Plaque Reduction Neutralization Test Titers After Secondary Infections to Assess Infecting Serotype

Comparing the models on the 86 observations at 6 months after secondary infection (follow-up 2), the PMA was better able to predict previous infecting serotype, compared with PRNT. The average test set model performance for PRNT in 3-fold cross-validation had κ = 0.09 and an accuracy of 0.35 (compared with κ = 0.27 and an accuracy of 0.47 for PMA). When only DENV antigens were included in the PMA model, the model had average κ = 0.06 and an accuracy of 0.28.

### Application of Model to Indeterminate Cases

To assess the application of our methods to samples from those classified as indeterminate by PRNT, we first applied models A (follow-up 1), B (follow-up 2), and C (both time points) to estimate the individuals’ immune status. The results generated were mostly consistent across time points, except for a few individuals. At both time points for model A and B, and at time point 1 only for model C, approximately 2 of 3 of patients were classified as having experienced a secondary infection, which is consistent with the acute titers [18] (Supplementary Figure S4). In model C, at follow-up time point 2, closer to 50% were classified as having experienced a secondary infection. In those individuals for which immune status classification by model C differed between time points, decreases in both DENV and non-DENV antibody titers between the 2 follow-up points were particularly noticeable.

For indeterminate individuals classified as having experienced a primary infection, we further applied model D to predict past infecting DENV serotype, and then we compared the predictions with RT-PCR results. At follow-up time point 1, the model performed well ([Supplementary Table S10] κ = 0.79, accuracy of 0.86). The model performed less well at follow-up time point 2 (κ = 0.55 and accuracy of 0.66) and at both time points (κ = 0.69 and accuracy of 0.78) (Supplementary Table S10). Due to the lower accuracy for predicting serotype after secondary infections, we did not attempt to classify the most recent infecting serotype for indeterminate cases classified as having experienced secondary infections using the PMA model.

## DISCUSSION

We assessed the applicability of a novel flavivirus microarray for use in seroepidemiological studies of dengue in endemic settings. We showed that the microarray was able to differentiate well between those who have experienced 1 or more than 1 past dengue infection (85% accuracy), and we correctly determine most recent past infecting serotype after primary infection (92% accuracy) but not after secondary (45% accuracy). This suggests that the assay with the models developed here would be of use to characterize past DENV transmission in seroepidemiological studies in DENV-endemic settings.

The difference in ability to estimate infecting serotype after 2 infections compared with after 1 infection should be expected, considering the well described cross-serotype antibody reactivity to dengue after 2 or more dengue infections and the process of antigenic sin [25–27]. At the 6-month time point, at which we were able to compare the accuracy of models using PMA versus PRNT measures to predict the second infecting serotype (defined by RT-PCR during acute infection), PMA did slightly better than the PRNT with an accuracy of 47% versus 35%, respectively. Our PRNT results are in good agreement with previous studies using PRNT to assess infection history in Thailand [28]. Comparison between the models using PMA with only DENV antigens versus DENV and non-DENV antigens shows that it is the inclusion of the non-DENV antigen titers in the model that gives the model with PMA higher accuracy than the model with DENV only PRNT. This suggests that after a second infection, infection with each serotypes generates differential cross-reactive responses to the different flaviviruses; specifically, these responses are more different than across the responses to the DENV serotypes. In our results, we saw that that cross-reactivity can, in some situations, be useful and tell us more about past transmission. The major benefit of the PMA assay is that the high-throughput nature means it is possible to test titers to multiple antigens at once, so these data can be generated more easily. In addition, PMA only requires a small amount of sera for testing against multiple antigens. The cost for PRNT and PMA are variable depending on the number of samples tested and where they are tested, and up-to-date costs should be considered when deciding on what assays to use in each situation.

We are also able to comment on the antibody responses after DENV infections to other flaviviruses. After a first dengue infection, we observed little flavivirus cross-reactivity, although more after a DENV2 infection. Dengue virus 2 infections were also much more likely to be classified as indeterminate by the PRNT than the other serotypes, suggesting perhaps that DENV2 infection elicits a different response upon first infection than the other serotypes. After a second infection, we observed high titers to all flaviviruses tested (WNV, SLEV, and ZIKV). St. Louis encephalitis virus and WNV are not thought to circulate in Vietnam; therefore, the positive responses after a second DENV infection are likely due to cross-reactivity. However, we know less about ZIKV infection in Vietnam, and further work will be needed assess whether there is any information about past ZIKV transmission in these titers. The DENV titers in our patient group were higher than the ZIKV titers, even after secondary infection, as has been seen elsewhere [29, 30]; therefore, if we are able to do the same study after ZIKV infection, or with well defined cohorts studying both infections (eg, [31, 32]), it is conceivable that cutoff using ratios of the titers could be defined for population-level exposure assessments for ZIKV and DENV in areas of cotransmission.

 Serological responses can be informative both about past transmission as well as immunity in the population. In this article, we do not draw any conclusions about what the titers mean for immunity, eg, whether it is protective or enhancing, because this requires functional antibody measurements for all viruses on the array. For dengue, the general consensus is that after 2 infections, individuals are protected against severe infection [33, 34], and the observed high titers measured in the PMA could be an indication of protection. However, caution is needed in interpreting these titers in terms of immunity.

There are a few extra considerations as we move forward to applying these models to infer past population transmission in population-level seroepidemiological studies. Age was included only in the best model for determining infecting serotype after secondary infections. This could be due to either a cohort effect in our samples or differences in how immunity develops with age. Further work in other settings or at different time points will be needed to determine which. In addition, we only tested samples from the age range that experience clinical disease, so age may not be possible to be used in future applications of this model. Our sensitivity analysis showed that the predicting infecting secondary serotype model performed slightly worse without age. We only tested samples up to 6 months after infection; however, the lack of change in titers between the 2 time points tested suggests that the models developed here could be applicable at later time points after 6 months postinfection, and from studies of PRNT after infection, we might expect only small further decrease in titers from 6 months to 1 year [25]. Nevertheless, this should continue to be assessed because these models are applied to population-level samples. In addition, we only tested samples in the highly endemic transmission setting of Vietnam; therefore, our models may need further testing in different transmission settings. However, we believe that if consistency is observed across multiple settings, these models could be applied to samples from dengue samples from any setting.

## CONCLUSIONS

In this study, we were able to develop predictive models using titer measures from a novel flavivirus PMA, and we showed that these should be useful for seroepidemiology studies by showing the models can (1) determine whether an individual has been infected with dengue once or more than once in the past and (2) determine the past infecting serotype for primary infections. These results, combined with the ease of performing the array and its replicability, suggest that it is an excellent candidate for use in dengue seroepidemiology studies. Our next step is to apply these models to population studies to infer past transmission in Vietnam where we will be able to assess the inferences made using these models at different ages and compare it with other measures of transmission. The use of the novel PMA and the models generated here will greatly facilitate further work on dengue epidemiology, which would help to shed light on the past transmission intensity in dengue-endemic populations.

## Supplementary Data

Supplementary materials are available at *The Journal of Infectious Diseases* online. Consisting of data provided by the authors to benefit the reader, the posted materials are not copyedited and are the sole responsibility of the authors, so questions or comments should be addressed to the corresponding author.

jiaa018_suppl_Supplementary_Information

## Notes

***Finnancial*** ***support*.** H. T. P., N. H. T. V., M. F. B., and H. E. C. acknowledge funding from a Wellcome Trust Enhancement Award (098511/Z/12/Z), and H. T. P., N. H. T. V., M. F. B., H. E. C., B. A. W., N. T. H. T., H. T. L. D., and D. T. T. acknowledge Wellcome Trust Centre funding to the Oxford University Clinical Research Unit. E. d. B. and M. K. acknowledge funding by ZIKALLIANCE. T. T. N. T. acknowledges funding from I&I Fund for Research Masters Infection and Immunity, Erasmus Postgraduate School of Molecular Medicine, and Erasmus University Holland Scholarship for non-European Union/European Economic Area students.

***Potential conflicts of interest*.** All authors: No reported conflicts of interest. All authors have submitted the ICMJE Form for Disclosure of Potential Conflicts of Interest.
