# Supplementary material for: Using NS1 Flavivirus Protein Microarray to Infer Past Infecting Dengue Virus Serotype and Number of Past Dengue Virus Infections in Vietnamese Individuals
Source: J Infect Dis. 2020 Jan 22;223(12):2053–61. doi: 10.1093/infdis/jiaa018 (PMC8205622; doi:10.1093/infdis/jiaa018)
Supplement: jiaa018_suppl_Supplementary_Information [file jiaa018_suppl_supplementary_information.pdf]

Supplementary Information

Commercially purchased recombinant flavivirus NS1 antigens (Table S1) were diluted to the desired concentration with protein arraying buffer (Main Manufacturing), and spotted onto a 64-pad nitrocellulose coated slide (Satorius, Germany), in duplicate, using a non-contact protein array spotter (Scienion, Germany). Each spot was a drop of 450 pL diluted protein.

SPOT POSITION	VIRUS	ANTIGEN	PRODUCT CODE	PRODUCT NAME
**1, 2**	Dengue virus - serotype 1	NS1	40527-V07H	NS1 (DV1) -DENV-NS1 (His Tag)
**3, 4**	Dengue virus - serotype 2	NS1	IT-006-0051p	NS1 (DENV2)
**5, 6**	Dengue virus - serotype 3	NS1	IT-006-0052p	NS1 (DENV3)
**7, 8**	Dengue virus - serotype 4	NS1	IT-006-0055p	NS1 (DENV4)
**9, 10**	West Nile virus	NS1	40346-V07H	West Nile Virus (WNV) (lineage 1, strain NY99) NS1 Protein (His Tag)
**11, 12**	Japanese Encephalitis virus	NS1	IT-006-054p	NS1 (JEV)
**13, 14**	Yellow fever virus	NS1	IT-006-0057p	NS1 (Yellow fever virus)
**15, 16**	Zika virus	NS1	IT-006-0059p	NS1 (Zika virus)
**17, 18**	Zika virus - Brasil strain	NS1	IT-006-0063p	NS1 (Zika virus/Brazil)
**19, 20**	St. Louis Encephalitis virus	NS1	IT-006-0058p	NS1 (St. Louis encephalitis virus)
**21, 22**	Chikungunya virus - mutant	E1	A2323	Rec. Chikungunya mutant E1 protein
**23, 24**	Chikungunya virus - mutant	E2	IT-022-004Ep	E2 (Chikungunya virus)
**25, 26**	Tick-borne Encephalitis virus	NS1	IT-006-0056P	NS1 (Tick-borne encephalitis virus)

Table S1. Information of flavivirus NS1 antigens included on one microarray glass slide.

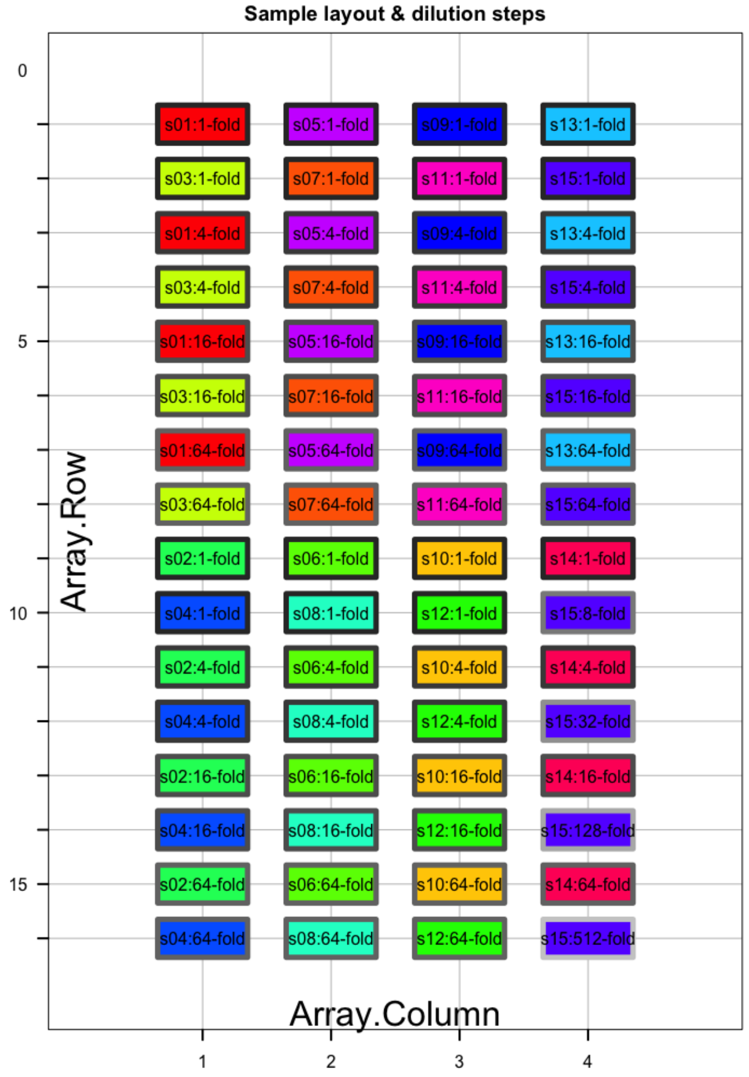
Sample layout & dilution steps
Array.Row
Array.Column
0
5
10
15
1
2
3
4
s01:1-fold
s05:1-fold
s09:1-fold
s13:1-fold
s03:1-fold
s07:1-fold
s11:1-fold
s15:1-fold
s01:4-fold
s05:4-fold
s09:4-fold
s13:4-fold
s03:4-fold
s07:4-fold
s11:4-fold
s15:4-fold
s01:16-fold
s05:16-fold
s09:16-fold
s13:16-fold
s03:16-fold
s07:16-fold
s11:16-fold
s15:16-fold
s01:64-fold
s05:64-fold
s09:64-fold
s13:64-fold
s03:64-fold
s07:64-fold
s11:64-fold
s15:64-fold
s02:1-fold
s06:1-fold
s10:1-fold
s14:1-fold
s04:1-fold
s08:1-fold
s12:1-fold
s15:8-fold
s02:4-fold
s06:4-fold
s10:4-fold
s14:4-fold
s04:4-fold
s08:4-fold
s12:4-fold
s15:32-fold
s02:16-fold
s06:16-fold
s10:16-fold
s14:16-fold
s04:16-fold
s08:16-fold
s12:16-fold
s15:128-fold
s02:64-fold
s06:64-fold
s10:64-fold
s14:64-fold
s04:64-fold
s08:64-fold
s12:64-fold
s15:512-fold

Figure S1. Layout of samples and dilutions tested on a microarray. (s = sample)

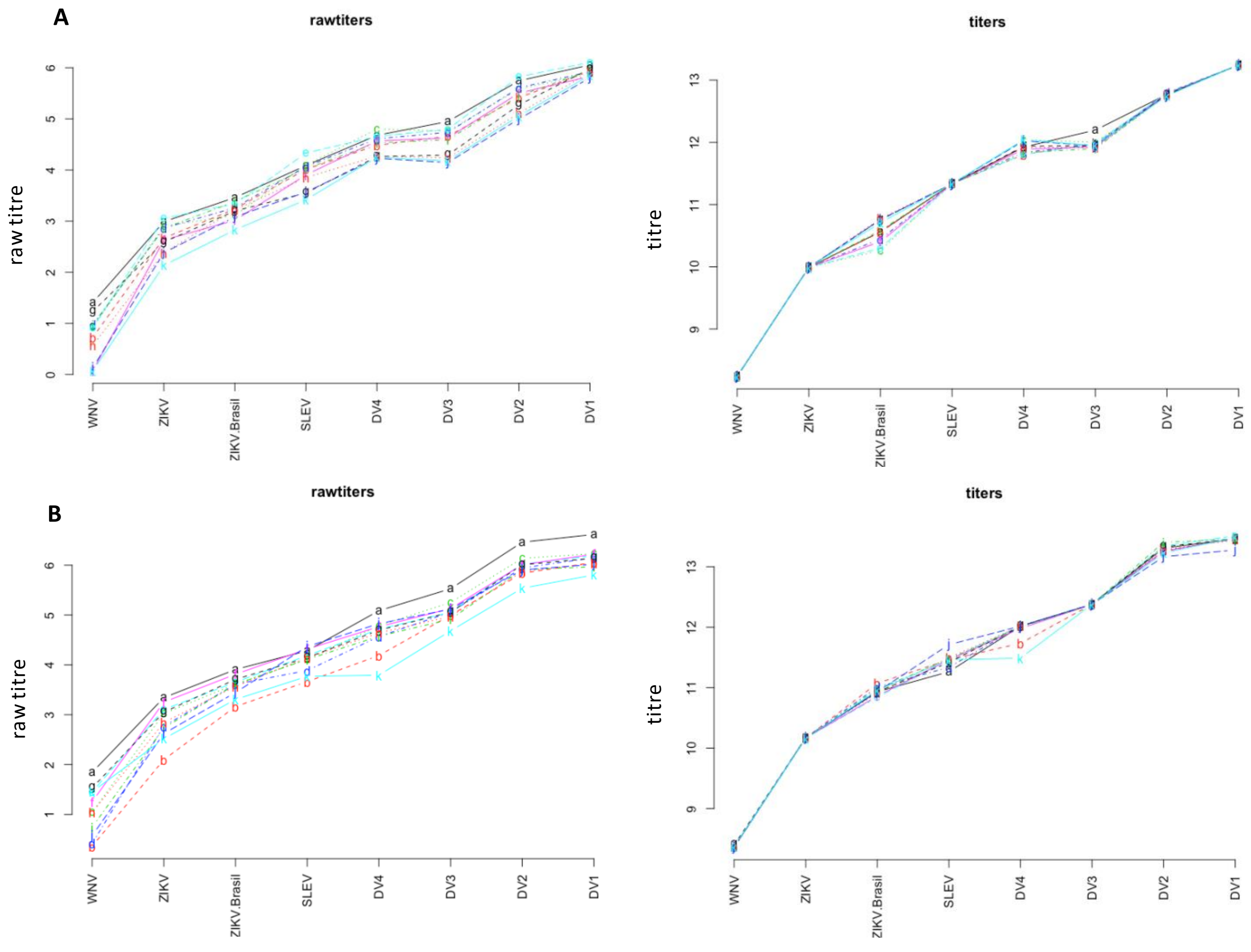

Figure S2. Before (left) and after (right) normalisation of positive controls included on tested microarray slides. x-axis indicates antigens that are included in analysis. y-axis of *rawtitres* plot indicates log2-transformed antibody titres without incorporating dilutions; and y-axis of *titres* plot indicates log2-transformed antibody titres after incorporating dilutions. (A) Quality control of the first batch including samples from hospital study; (B) Quality control of the second batch including samples from community study.

Figure S3. Changes in microarray titres between two follow-up points. In each follow-up, a dot represents antibody titre of one sample.

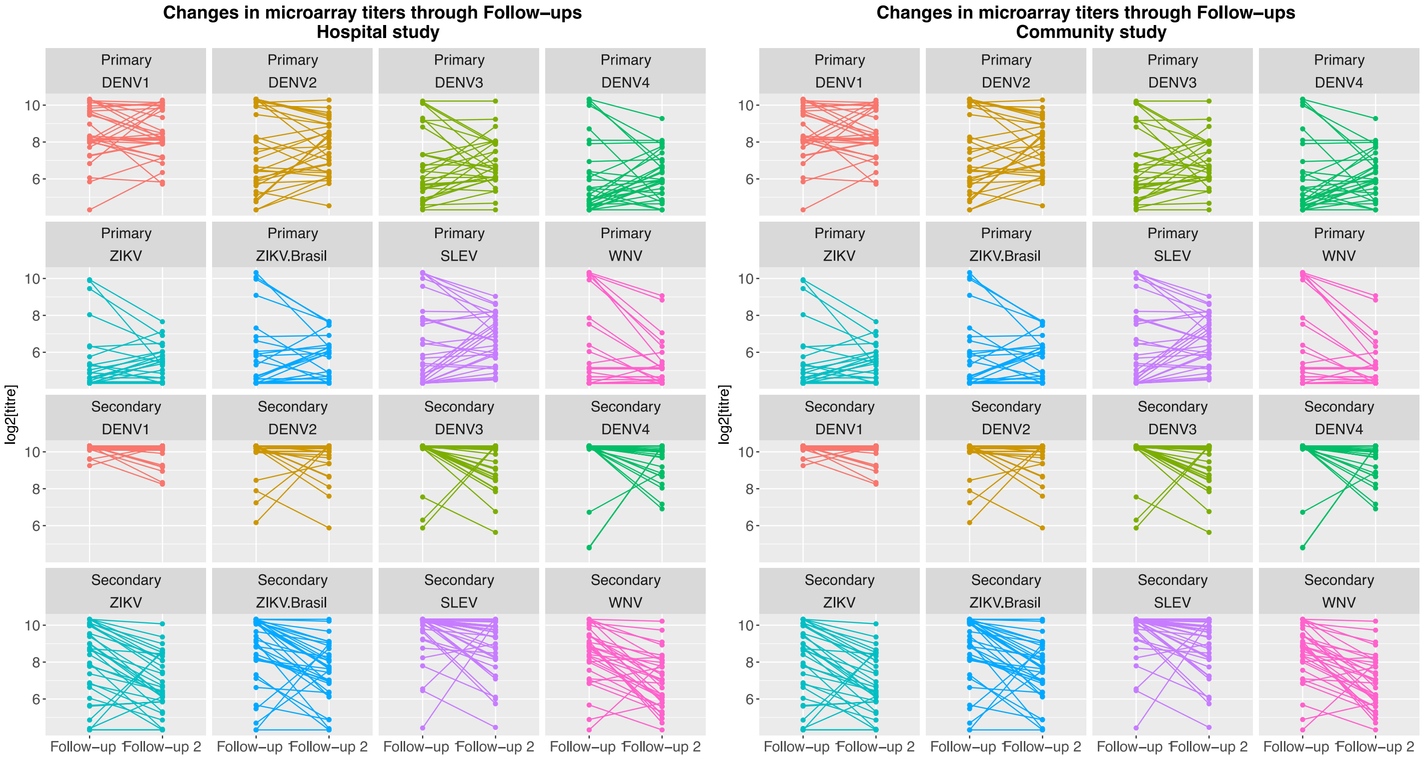

		Both follow-ups		Follow-up 1		Follow-up 2	
Outcome	Variable	Estimate	p-value	Estimate	p-value	Estimate	p-value
DENV1 titre	Age	-0.141	0.932	-0.042	0.988	1.315	0.492
	Gender (Female)	-0.011	0.945	-0.160	0.520	-0.069	0.247
	Months to follow-ups	0.033	0.126	0.933	0.064	-0.069	0.247
	Immune Status (Secondary)	2.047	<0.001	2.376	<0.001	1.642	<0.001
	Immune Status * Months to follow-up	-0.090	0.041	-2.108	0.024	-0.040	0.760
	Serotype 1	8.242	<0.001	7.373	<0.001	8.918	<0.001
	Serotype 2	7.861	<0.001	7.064	<0.001	8.425	<0.001
	Serotype 3	7.163	<0.001	5.933	<0.001	8.206	<0.001
	Serotype 4	7.936	<0.001	7.218	<0.001	8.548	<0.001
DENV2 titre	Age	3.552	0.035	2.938	0.289	-0.069	0.247
	Gender (Female)	-0.106	0.494	-0.272	0.273	0.063	0.737
	Months to follow-ups	0.037	0.096	0.209	0.676	-0.052	0.415
	Immune Status (Secondary)	2.114	<0.001	2.390	<0.001	1.786	<0.001
	Immune Status * Months to follow-up	-0.078	0.083	-0.482	0.611	0.057	0.686
	Serotype 1	6.794	<0.001	6.507	<0.001	7.585	<0.001
	Serotype 2	7.919	<0.001	7.955	<0.001	8.327	<0.001
	Serotype 3	6.458	<0.001	6.021	<0.001	7.345	<0.001
	Serotype 4	7.346	<0.001	7.322	<0.001	7.821	<0.001
DENV3 titre	Age	1.367	0.465	3.109	0.319	0.732	0.742
	Gender (Female)	0.048	0.778	-0.061	0.828	0.211	0.302
	Months to follow-ups	0.005	0.833	0.644	0.254	-0.082	0.234
	Immune Status (Secondary)	2.942	<0.001	3.204	<0.001	2.618	<0.001
	Immune Status * Months to follow-up	-0.078	0.119	-0.528	0.620	0.064	0.675
	Serotype 1	6.294	<0.001	5.355	<0.001	7.212	<0.001

	**Serotype 2**	6.739	<0.001	6.052	<0.001	7.366	<0.001
	**Serotype 3**	7.177	<0.001	5.862	<0.001	8.466	<0.001
	**Serotype 4**	6.924	<0.001	6.208	<0.001	7.667	<0.001
**DENV4 titre**	**Age**	0.767	0.714	2.519	0.486	0.515	0.831
	**Gender (Female)**	0.102	0.596	0.148	0.648	0.135	0.541
	**Months to follow-ups**	0.010	0.712	0.882	0.179	-0.070	0.346
	**Immune Status (Secondary)**	3.286	<0.001	3.448	<0.001	3.060	<0.001
	**Immune Status * Months to follow-up**	-0.044	0.428	0.310	0.802	0.331	0.050
	**Serotype 1**	5.860	<0.001	4.738	<0.001	6.652	<0.001
	**Serotype 2**	6.436	<0.001	5.522	<0.001	6.998	<0.001
	**Serotype 3**	5.406	<0.001	4.091	<0.001	6.414	<0.001
	**Serotype 4**	7.029	<0.001	6.098	<0.001	7.735	<0.001
**SLEV titre**	**Age**	6.420	0.004	7.135	0.045	6.755	0.017
	**Gender (Female)**	-0.106	0.603	-0.156	0.623	-0.006	0.982
	**Months to follow-ups**	-0.036	0.219	0.571	0.372	-0.120	0.167
	**ImmuneStatus (Secondary)**	2.700	<0.001	3.105	<0.001	2.235	<0.001
	**ImmuneStatus * Months to follow-up**	-0.148	0.011	-0.757	0.531	0.020	0.921
	**Serotype 1**	5.747	<0.001	4.764	<0.001	6.717	<0.001
	**Serotype 2**	6.297	<0.001	5.631	<0.001	6.914	<0.001
	**Serotype 3**	5.327	<0.001	4.110	<0.001	6.524	<0.001
	**Serotype 4**	5.839	<0.001	5.549	<0.001	6.161	<0.001
**WNV titre**	**Age**	4.306	0.064	6.924	0.060	2.736	0.373
	**Gender (Female)**	-0.011	0.959	-0.049	0.882	0.109	0.699
	**Months to follow-ups**	-0.162	<0.001	0.474	0.474	-0.072	0.452
	**ImmuneStatus (Secondary)**	2.151	<0.001	2.468	<0.001	1.862	<0.001
	**ImmuneStatus * Months to follow-up**	-0.103	0.093	-0.734	0.557	0.053	0.802

	**Serotype 1**	5.564	<0.001	4.496	<0.001	5.223	<0.001
	**Serotype 2**	5.973	<0.001	5.044	<0.001	5.592	<0.001
	**Serotype 3**	5.095	<0.001	3.747	<0.001	5.173	<0.001
	**Serotype 4**	5.717	<0.001	5.014	<0.001	5.241	<0.001
**ZIKV titre**	**Age**	7.047	0.003	5.103	0.173	8.062	0.008
	**Gender (Female)**	-0.180	0.402	-0.312	0.352	-0.099	0.717
	**Months to follow-ups**	-0.095	0.002	-0.642	0.342	-0.066	0.479
	**ImmuneStatus (Secondary)**	2.605	<0.001	3.176	<0.001	2.066	<0.001
	**ImmuneStatus * Months to follow-up**	-0.176	0.004	-1.353	0.288	0.009	0.964
	**Serotype 1**	4.903	<0.001	5.296	<0.001	4.880	<0.001
	**Serotype 2**	4.886	<0.001	5.311	<0.001	4.826	<0.001
	**Serotype 3**	4.414	<0.001	4.593	<0.001	4.571	<0.001
	**Serotype 4**	4.818	<0.001	5.526	<0.001	4.389	<0.001
**ZIKV.Brasil titre**	**Age**	6.056	0.010	4.671	0.207	7.026	0.022
	**Gender (Female)**	-0.160	0.457	-0.338	0.308	-0.017	0.953
	**Months to follow-ups**	-0.091	0.004	-0.347	0.604	-0.140	0.137
	**ImmuneStatus (Secondary)**	2.868	<0.001	3.455	<0.001	2.263	<0.001
	**ImmuneStatus * Months to follow-up**	-0.185	0.002	-1.512	0.230	0.020	0.922
	**Serotype 1**	5.195	<0.001	5.226	<0.001	5.831	<0.001
	**Serotype 2**	5.432	<0.001	5.612	<0.001	5.869	<0.001
	**Serotype 3**	4.805	<0.001	4.532	<0.001	5.678	<0.001
	**Serotype 4**	5.179	<0.001	5.647	<0.001	5.269	<0.001

Table S2. Multivariable linear regression analysis. In each regression, we examined the effects of multiple variables (i.e. age, gender as female, immune status as being a secondary infection, and infecting DENV serotype) on an outcome (i.e. microarray titre). Note that 'Age' was scaled by dividing the values by 100, and 'Months to follow-ups' was scaled by dividing 'Days to follow-ups' by 30.

Data	Outcome	Predictors	AIC
Follow-up 1	Immune status	DENV1 + DENV2 + DENV3 + DENV4 + WNV + ZIKV + ZIKV.Brasil + SLEV + Age	108.53
		DENV1 + DENV2 + DENV3 + DENV4 + WNV + ZIKV + SLEV + Age	106.59
		DENV1 + DENV3 + DENV4 + WNV + ZIKV + SLEV + Age	104.7
		DENV3 + DENV4 + WNV + ZIKV + SLEV + Age	103.12
		DENV3 + DENV4 + WNV + ZIKV + SLEV	101.74
		DENV3 + DENV4 + ZIKV + SLEV	101.61

Data	Outcome	Predictors	AIC
Follow-up 2	Immune status	DENV1 + DENV2 + DENV3 + DENV4 + WNV + ZIKV + ZIKV.Brasil + SLEV + Age	86.6
		DENV1 + DENV2 + DENV3 + DENV4 + WNV + ZIKV + ZIKV.Brasil + SLEV	84.6
		DENV1 + DENV3 + DENV4 + WNV + SLEV	80.67
		DENV3 + DENV4 + WNV + ZIKV + SLEV	78.92
		DENV3 + DENV4 + ZIKV + SLEV	78.04

Data	Outcome	Predictors	AIC
Both follow-ups	Immune status	DENV1 + DENV2 + DENV3 + DENV4 + WNV + ZIKV + ZIKV.Brasil + SLEV + Age	188.16
		DENV1 + DENV3 + DENV4 + WNV + ZIKV + ZIKV.Brasil + SLEV	186.18
		DENV1 + DENV3 + DENV4 + WNV + ZIKV + SLEV + Age	182.49
		DENV3 + DENV4 + WNV + ZIKV + SLEV	180.91

Table S3.AIC for all models considered for determining immune status

Model	Test set	
**Model A (follow-up point 1)**<br>**(141 observations: 55 primary cases and 86 secondary cases)**	**AUC (95% CI)**	**Accuracy (95% CI)**
**Round 1**	0.97 (0.91-1)	0.90 (0.73-0.98)
**Round 2**	0.82 (0.60-1)	0.71 (0.51-0.87)
**Round 3**	0.92 (0.80-1)	0.82 (0.63-0.94)
**Round 4**	0.88 (0.72-0.99)	0.82 (0.63-0.94)
**Round 5**	1 (1-1)	0.96 (0.82-1)
**Average**	0.90 (0.83-0.96)	0.84 (0.66-0.75)
**Model B (follow-up point 2)**<br>**(141 observations: 55 primary cases and 86 secondary cases)**	**AUC (95% CI)**	**Accuracy (95% CI)**
**Round 1**	0.93 (0.81-1)	0.86 (0.68-0.96)
**Round 2**	0.96 (0.89-1)	0.82 (0.63-0.94)
**Round 3**	0.97 (0.91-1)	0.89 (0.72-0.98)
**Round 4**	0.94 (0.84-1)	0.93 (0.77-0.99)
**Round 5**	0.96 (0.89-1)	0.82 (0.63-0.94)
**Average**	0.95 (0.92-0.98)	0.86 (0.68-0.96)
**Model C (both follow-up points)**<br>**(282 observations: 55 primary cases and 86 secondary cases)**	**AUC (95% CI)**	**Accuracy (95% CI)**
**Round 1**	0.93 (0.85-0.98)	0.82 (0.70-0.91)
**Round 2**	0.93 (0.86-0.99)	0.86 (0.74-0.94)
**Round 3**	0.95 (0.88-0.99)	0.84 (0.72-0.93)
**Round 4**	0.94 (0.86-0.99)	0.89 (0.78-0.96)
**Round 5**	0.92 (0.84-0.98)	0.84 (0.72-0.93)
**Average**	0.93 (0.90-0.96)	0.85 (0.73-0.93)

Table S4: Results for each round of 5-fold validation for the models determining immune status

**Model**	**Test set**	
**Model D (primary infections)**<br>**(110 observations: 28 DENV1, 17 DENV2, 7 DENV3, 3 DENV4)**	**Kappa (95% CI)**	**Accuracy (95% CI)**
**Round 1**	0.83 (0.67-0.98)	0.89 (0.75-0.97)
**Round 2**	0.92 (0.80-1)	0.95 (0.82-0.99)
**Round 3**	0.86 (0.71-1)	0.92 (0.78-0.98)
**Average**	0.87 (0.72-0.99)	0.92 (0.78-0.98)
**Model E (secondary infections)**<br>**(172 observations: 27 DENV1, 26 DENV2, 12 DENV3, 21 DENV4)**	**Kappa (95% CI)**	**Accuracy (95% CI)**
**Round 1**	0.24 (0.10-0.42)	0.46 (0.32-0.59)
**Round 2**	0.31 (0.14-0.48)	0.49 (0.36-0.63)
**Round 3**	0.16 (0-0.33)	0.40 (0.27-0.53)
**Average**	0.24 (0.08-0.41)	0.45 (0.32-0.58)
**Model F (including secondary infections at higher dilutions)**<br>**(172 observations: 27 DENV1, 26 DENV2, 12 DENV3, 21 DENV4)**	**Kappa (95% CI)**	**Accuracy (95% CI)**
**Round 1**	0.14 (-0.03, 0.31)	0.36 (0.34-0.61)
**Round 2**	0.27 (0.12, 0.41)	0.43 (0.30, 0.57)
**Round 3**	0.35 (0.18, 0.52)	0.52 (0.39, 0.66)
**Average**	0.25 (0.09, 0.41)	0.43 (0.34, 0.61)

Table S5: Results for each round of 3-fold validation for the models determining most recent infecting serotype

	Model A				Model B				Model C			
Round 1			True				True				True	
			Primary	Secondary			Primary	Secondary			Primary	Secondary
	Predicted	Primary	9	1	Predicted	Primary	10	3	Predicted	Primary	19	7
		Secondary	2	17		Secondary	1	15		Secondary	3	27
Round 2			True				True				True	
			Primary	Secondary			Primary	Secondary			Primary	Secondary
	Predicted	Primary	8	5	Predicted	Primary	10	4	Predicted	Primary	18	4
		Secondary	3	12		Secondary	1	13		Secondary	4	30
Round 3			True				True				True	
			Primary	Secondary			Primary	Secondary			Primary	Secondary
	Predicted	Primary	9	3	Predicted	Primary	10	2	Predicted	Primary	21	8
		Secondary	2	14		Secondary	1	15		Secondary	1	27
Round 4			True				True				True	
			Primary	Secondary			Primary	Secondary			Primary	Secondary
	Predicted	Primary	10	4	Predicted	Primary	10	1	Predicted	Primary	19	3
		Secondary	1	13		Secondary	1	16		Secondary	3	31
Round 5			True				True				True	
			Primary	Secondary			Primary	Secondary			Primary	Secondary
	Predicted	Primary	10	0	Predicted	Primary	11	5	Predicted	Primary	21	8
		Secondary	1	17		Secondary	0	12		Secondary	1	27

Table S6. Confusion matrices of immune status defined by PRNT60 (**True**), and by predictive binomial logistic models A, B and C (**Predicted**) when applying the models on test sets in 5-fold cross-validation.

	Model A				Model B				Model C			
	**SENS**	**SPEC**	**PPV**	**NPV**	**SENS**	**SPEC**	**PPV**	**NPV**	**SENS**	**SPEC**	**PPV**	**NPV**
**Round 1**	0.82	0.94	0.9	0.89	0.90	0.83	0.77	0.94	0.86	0.79	0.73	0.9
**Round 2**	0.73	0.71	0.62	0.80	0.91	0.76	0.71	0.93	0.82	0.88	0.82	0.88
**Round 3**	0.82	0.82	0.75	0.88	0.91	0.88	0.83	0.94	1	0.77	0.73	1
**Round 4**	0.91	0.76	0.71	0.93	0.91	0.94	0.91	0.94	0.91	0.91	0.87	0.94
**Round 5**	0.91	1	1.00	0.94	0.91	0.76	0.71	0.93	0.77	0.80	0.71	0.85

Table S7. Additional metrics illustrating the performance of predictive binomial logistic models A, B and C on test sets in 5-fold cross validation. **SENS:** sensitivity, **SPEC:** specificity, **PPV:** positive predictive value, **NPV:** negative predictive value.

			Model D				Model E				Model F			
Round 1			True				True				True			
			DENV1	DENV2	DENV3	DENV4	DENV1	DENV2	DENV3	DENV4	DENV1	DENV2	DENV3	DENV4
	Predicted	DENV1	18	0	0	0	11	3	2	4	5	2	2	4
		DENV2	2	9	1	0	8	8	2	5	11	7	3	4
		DENV3	1	0	4	0	0	0	3	1	1	1	3	0
		DENV4	1	1	0	2	3	2	1	4	4	4	0	6
Round 2			True				True				True			
			DENV1	DENV2	DENV3	DENV4	DENV1	DENV2	DENV3	DENV4	DENV1	DENV2	DENV3	DENV4
	Predicted	DENV1	15	0	0	0	12	4	2	4	11	15	2	6
		DENV2	2	13	0	0	1	5	0	3	0	3	3	0
		DENV3	1	0	6	0	1	1	4	0	1	4	3	0
		DENV4	0	0	0	1	2	9	2	7	0	2	0	8
Round 3			True				True				True			
			DENV1	DENV2	DENV3	DENV4	DENV1	DENV2	DENV3	DENV4	DENV1	DENV2	DENV3	DENV4
	Predicted	DENV1	19	0	1	0	9	8	3	5	9	4	1	0
		DENV2	0	11	0	2	4	6	3	2	7	8	4	3
		DENV3	0	0	2	0	1	0	1	0	0	1	2	0
		DENV4	0	0	0	1	2	6	1	7	5	1	1	11

Table S8. Confusion matrices of infecting DV serotype defined by RT-PCR (**True**), and by predictive multinomial logistic models D, E and F (**Predicted**) when applying the models on test sets in 3-fold cross-validation.

		Model D				Model E				Model F			
		**SENS**	**SPEC**	**PPV**	**NPV**	**SENS**	**SPEC**	**PPV**	**NPV**	**SENS**	**SPEC**	**PPV**	**NPV**
**Round 1**	**DENV1**	0.90	1	1	0.89	0.50	0.74	0.55	0.70	0.23	0.77	0.38	0.63
	**DENV2**	0.90	0.89	0.75	0.96	0.62	0.66	0.38	0.5	0.50	0.58	0.28	0.78
	**DENV3**	0.80	1	1	0.97	0.38	0.98	0.75	0.91	0.37	0.95	0.60	0.90
	**DENV4**	1	0.97	0.67	1	0.29	0.86	0.40	0.79	0.42	0.81	0.43	0.81
**Round 2**	**DENV1**	0.88	1	1	0.91	0.75	0.76	0.55	0.89	0.91	0.12	0.38	0.57
	**DENV2**	1	0.92	0.87	1	0.26	0.89	0.56	0.71	0.50	0.91	0.90	0.95
	**DENV3**	1	1	1	1	0.5	0.96	0.7	0.92	0.37	0.90	0.37	0.90
	**DENV4**	1	1	1	1	0.5	0.70	0.35	0.81	0.57	0.95	0.80	0.87
**Round 3**	**DENV1**	1	0.94	0.95	1	0.56	0.62	0.36	0.79	0.42	0.86	0.64	0.72
	**DENV2**	1	0.92	0.85	1	0.3	0.76	0.40	0.67	0.57	0.67	0.36	0.82
	**DENV3**	0.67	1	1	0.97	0.13	0.98	0.50	0.88	0.25	0.97	0.66	0.88
	**DENV4**	0.33	1	1	0.94	0.50	0.80	0.44	0.83	0.78	0.83	0.61	0.92

Table S9. Additional metrics illustrating the performance of predictive binomial logistic models D, E and F on test sets 3-fold cross validation. **SENS:** sensitivity, **SPEC:** specificity, **PPV:** positive predictive value, **NPV:** negative predictive value.

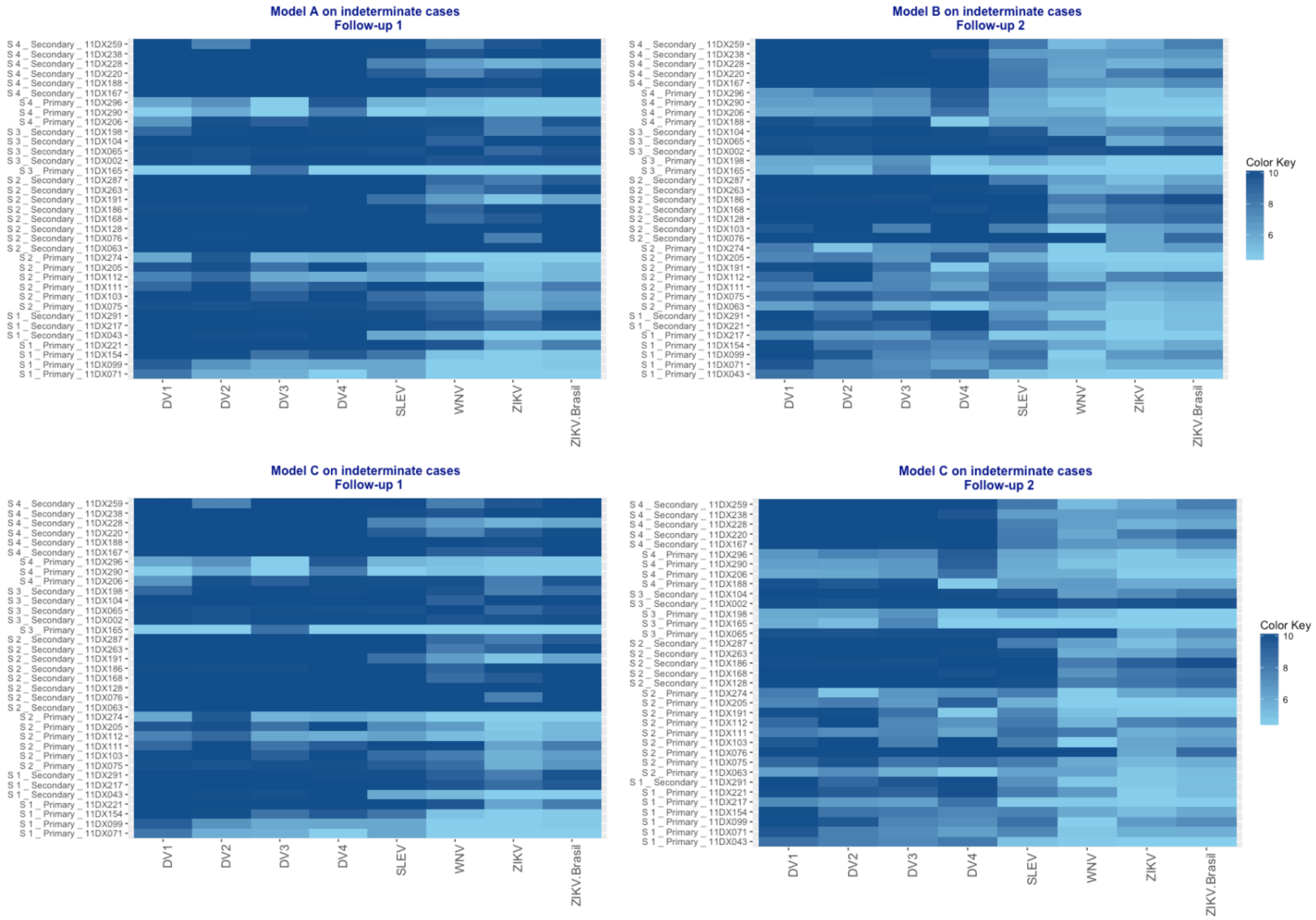

Figure S4. **Heatmaps illustrating antibody profiles of indeterminate cases at two follow-up time points (S: serotype).**

Infecting DV serotype of predicted primary cases by model A		Predicted				Kappa (95% CI)	Accuracy (95% CI)
		DV1	DV2	DV3	DV4		
True	DENV1	3	1	0	0	0.79 (0.52-1)	0.86 (0.57-0.98)
	DENV2	1	5	0	0		
	DENV3	0	0	1	0		
	DENV4	0	0	0	3		
Infecting DV serotype of predicted primary cases by model B		Predicted				Kappa (95% CI)	Accuracy (95% CI)
		DV1	DV2	DV3	DV4		
True	DENV1	4	3	0	1	0.55 (0.26-0.84)	0.66 (0.41-0.87)
	DENV2	0	3	0	0		
	DENV3	0	0	2	0		
	DENV4	1	1	0	3		
Infecting DV serotype of predicted primary cases by model C		Predicted				Kappa (95% CI)	Accuracy (95% CI)
		DV1	DV2	DV3	DV4		
True	DENV1	8	4	0	1	0.69 (0.50-0.88)	0.78 (0.61-0.90)
	DENV2	1	10	0	0		
	DENV3	0	0	4	0		
	DENV4	1	1	0	6		

Table S10. **Results of serotype-identifying model D when applied on indeterminate cases that have been classified as primary cases by models A, B and C. The table includes confusion matrices of infecting DV serotype defined by RT-PCR (True) and by model D (Predicted), and performance of model D assessed by kappa statistic and accuracy.**

User-defined R scripts
These use-defined R scripts should be used in the following order. With a description of what each file does. Files after titre calculation are made available as an appendix to the paper. Raw files of slides are available on request (large files).

1. **Titre calculation**
    a. **Calculation of the PMA titre that is used in the analysis from the raw scanned data files including positive control**
    b. **Performing quality control on the obtained titres**
2. **Model selection and validation (after titre calculation)**
    a. **With the titres obtained in section 1 the different models were tested using cross-validation for i) predicting the immune status of patients, ii) predicting the serotype of infections for each immune status group**

Scripts

1. **Titre calculation**

Create a modified 64-pad microarray layout. This is the layout in which 1 positive control is tested in 8 dilutions.

```
# Load normal layout
plotArrayLayout(layout64pad4x4fold)

# Modify the loaded layout
newlayout = layout64pad4x4fold
newlayout[newlayout$agArrayID == 's16', 'DilutionSteps'] = c(8,32,128,512)
newlayout[newlayout$agArrayID == 's16', 'agArrayID'] = rep('s15',4)

# Load the new layout
plotArrayLayout(newlayout)
```

Prepare data for titre calculation.

```
# Import files containing fluorescent score data read from software
arrayList = readArrayFiles(directory = './files', array.layout = newlayout)

# Import worklist file
meta = readWorklist('./files/worklist.txt')

# Remove 's16', because we have 1 positive control tested in 8 dilutions
meta2 = meta[-grep('s16', meta$agArrayID), ]
```

Titre calculation.

# Calculate titres using Median fluorescent values. Automatic normalisation is performed if positive control label is indicated

```
agset = createAgArraySet(meta2, agArrayList = arrayList, pos.contr.label = 'PC', exclude.antigens = 'Empty', Value = 'Median')
```

Quality Control.

# Plotting of positive controls is meant for checking slides with strangely low or high values. If there is any, remove it, then perform normalisation and titre calculation again.

```
plotPositiveControl(agset, 'PC', what = 'titers')
```

# Clean Antigens - There is a number of antigens against which positive control only gave background values. Normalisation cannot perform properly on background values. Therefore, these 'background' antigens will be removed.

```
bg.antigens = c("CHIKV E1 mutant", "CHIKV E2", "TBEV - NS1", "YFV - NS1", "JEV - NS1", 'Empty')

agset1 = createAgArraySet(meta2, agArrayList=arrayList, pos.contr.label = 'PC', exclude.antigens = bg.antigens, Value = 'Median')

plotPositiveControl(agset1, 'PC', what = 'titers')
```

2. Model selection and validation

Generation of training set and test set for 5-fold cross validation (3-fold was similarly done).

# Stratified sampling to maintain primary/secondary proportions as in the original dataset.
set.seed (1)
folds = createFolds(data$ImmuneStatus, k=5, list=T, returnTrain=F)

# Assigning of folds as training set and test set.
## In the first round, fold 1 will be assigned as test set. The remaining folds will be used as training set.
names(folds)[1] = 'test'
names(folds)[2] = 'train1'
names(folds)[3] = 'train2'
names(folds)[4] = 'train3'
names(folds)[5] = 'train4'

train = data.frame(rbind(data[folds$train1,], data[folds$train2,], data [folds$train3,], data [folds$train4,]))
test = data.frame(data [folds$test,])

Model performance on training set.

Binomial logistic models A, B and C predicting immune status.

# Step-wise backward elimination approach starts with an all-inclusive models containing age and microarray titres measured at:

## Follow-up 1
step(glm(ImmuneStatus ~ DV1 + DV2 + DV3 + DV4 + WNV + ZIKV + ZIKV.Brasil + SLEV + Age, data = data.fu1, family = binomial))

## Follow-up 2
step(glm(ImmuneStatus ~ DV1 + DV2 + DV3 + DV4 + WNV + ZIKV + ZIKV.Brasil + SLEV + Age, data = data.fu2, family = binomial))

## Both follow-ups
step(glm(ImmuneStatus ~ DV1 + DV2 + DV3 + DV4 + WNV + ZIKV + ZIKV.Brasil + SLEV + Age, data = data, family = binomial))

Multinomial logistic models D and E predicting infecting dengue serotype.

# Step-wise backward elimination approach starts with an all-inclusive models containing age and microarray titres of:

## Primary cases
step(multinom(Serotype ~ DV1 + DV2 + DV3 + DV4 + SLEV + WNV + ZIKV + ZIKV.Brasil + Age, data = primary))

```
    ## Secondary cases
step(multinom(Serotype ~ DV1 + DV2 + DV3 + DV4 + SLEV + WNV + ZIKV + ZIKV.Brasil + Age, data = secondary))

# Area under the ROC curve (AUC) of model fitting on training set.
roc.MP.1 = roc(train$ImmuneStatus, lgr$fitted.values)
roc.MP.1$auc
    ## 95% confidence interval of AUC
set.seed(11)
ci.auc(roc.MP.1, conf.level = 0.95, method = 'bootstrap', boot.n = 2000)
```

Model performance on test set.

```
# Model prediction given the test set.
prob.MV.1 = as.vector(predict.glm(lgr, newdata = test, type = 'response'))

# Area under the ROC curve (AUC) of model predicting on test set.
roc.MV.1= roc(test$ImmuneStatus, prob.MV.1)
    ## 95% confidence interval of AUC
set.seed(11)
ci.auc(roc.MV.1, conf.level = 0.95, method = 'bootstrap', boot.n = 2000)
```
